# Effects of biodiversity on functional stability of freshwater wetlands: a systematic review

**DOI:** 10.3389/fmicb.2024.1397683

**Published:** 2024-04-08

**Authors:** Aiwen Song, Shen Liang, Huai Li, Baixing Yan

**Affiliations:** ^1^State Key Laboratory of Black Soils Conservation and Utilization, Northeast Institute of Geography and Agroecology, Chinese Academy of Sciences, Changchun, China; ^2^University of Chinese Academy of Sciences, Beijing, China

**Keywords:** biodiversity, habitat functional stability, freshwater wetlands, habitat change, impact mechanisms

## Abstract

Freshwater wetlands are the wetland ecosystems surrounded by freshwater, which are at the interface of terrestrial and freshwater ecosystems, and are rich in ecological composition and function. Biodiversity in freshwater wetlands plays a key role in maintaining the stability of their habitat functions. Due to anthropogenic interference and global change, the biodiversity of freshwater wetlands decreases, which in turn destroys the habitat function of freshwater wetlands and leads to serious degradation of wetlands. An in-depth understanding of the effects of biodiversity on the stability of habitat function and its regulation in freshwater wetlands is crucial for wetland conservation. Therefore, this paper reviews the environmental drivers of habitat function stability in freshwater wetlands, explores the effects of plant diversity and microbial diversity on habitat function stability, reveals the impacts and mechanisms of habitat changes on biodiversity, and further proposes an outlook for freshwater wetland research. This paper provides an important reference for freshwater wetland conservation and its habitat function enhancement.

## 1 Introduction

Freshwater wetlands (FWs) are ecosystems formed by the interaction between freshwater rivers, lakes and land, mainly including riverine wetlands, lakes, marshes and floodplains. FWs not only provide suitable habitats for many plants and animals (McKown et al., 2021), but also play an important role in nutrient cycling, water purification and biodiversity maintenance (Li C. et al., 2022; Yu et al., 2023; Li et al., 2024). FWs have four the ecological services categories: provisioning, regulating, cultural and supporting services (Keddy et al., 2009). However, FWs have been severely damaged due to the increase in global population and economic development, resulting in a decrease in the global wetland area (Davidson, 2014), and a consequent severe destruction of wetland functions and biodiversity (Herbert et al., 2015; Ndehedehe et al., 2020).

Biodiversity is a complex system formed by the interaction between organisms and the external environment, expressing in genetic diversity, species diversity, and ecosystem diversity (Song, 2017; Liang et al., 2023). Habitat function refers to the specific functions and conditions providing for organisms, and many studies have shown that biodiversity plays a crucial role in habitat function and its stability (Weisser et al., 2017; Yao et al., 2017). FWs are complex ecosystems composed of special environmental conditions and organisms, and their functional stability is affected by many factors (Rideout et al., 2022). In FWs, high biodiversity can enhance the stability of wetland functions, such as nutrient cycling, water purification, and biodiversity maintenance (Thomaz, 2023). Rich diversity can alleviate competitive pressures among organisms by providing more ecological niches through complementary effects, allowing different species in FWs to fully utilize resources such as water, nutrients and sunlight (Steudel et al., 2011). In addition, biodiversity can also improve the stability and disturbance resistance of food chains, mitigating external disturbances in wetlands by building complex foodweb structures (Peel et al., 2019; Hatton et al., 2024).

Although many studies showed that the biodiversity of FWs has an important impact on the functional stability of the habitats in which they exist, few literatures have been reviewed and summarized. Therefore, the objectives of this study are to (1) analyze the effects of biodiversity on the functional stability of freshwater wetland habitats; (2) illuminate the impacts and mechanisms of habitat change on biodiversity; and (3) propose future research directions and perspectives. This paper synthesizes the environmental drivers of functional stability in FWs, the effects of plant and microbial diversity on the functional stability of FWs, and further discusses the effects and mechanisms of habitat change on biodiversity.

## 2 Environmental drivers of functional stability in freshwater wetlands

Freshwater wetlands provide numerous functions such as biodiversity maintenance, freshwater supply, carbon storage, etc., and at the same time they are one of the most fragile ecosystems (Zedler and Kercher, 2005). Changes in environmental drivers such as hydrological factors, climatic factors, water quality, and soil physicochemical properties have led to serious functional degradation of some wetlands (Xue et al., 2018; Xiu et al., 2019). Therefore, understanding the effects of these environmental drivers on freshwater wetland ecosystems (Table 1) is important for improving the functional stability of FWs and optimizing wetland management options.

**Table 1:** Effects of environmental drivers on freshwater wetland ecosystems.

Environmental drivers	Factor change	Functional changes	References
Wetland hydrology: Lake levels, rainfall, runoff, land use, and groundwater recharge	Extreme water level	Reduced primary productivity of wetlands.	Ojdanič et al. (2023)
	Lowering of wetland levels	Reduced biodiversity and biomass simplifies wetland function.	Gutierrez et al. (2022)
	Excessive high/low groundwater levels	Wetland water levels affect biogeochemical cycles, increasing CO and NO emissions when water is low and CH_4_ emissions when water is high.	Yang et al. (2013)
	Lowering of lake and river levels	Wetland habitat suitability declines, negatively affecting wetland functions.	Mu et al. (2022)
Physicochemical indicators: Water quality indicators: pH, DO, nutrient salt content, etc. Soil indicators: soil texture, organic matter content, pH, etc.	Wetland salinization	Water chemistry changes negatively impact biological activity and ecological processes in wetlands.	Herbert et al. (2015)
	Increased nutrient salts in the water column	Eutrophication of water bodies; changes in microbial communities and primary productivity.	Donato et al. (2020)
	Increased quality and availability of soil organic matter	Altered microbial community structure and increased wetland CO_2_ and CH_4_ production rates.	Morrissey et al. (2014)
	Reduced pH of wetland soils	Changes in the structure of microbial communities negatively impact the functions of wetlands.	Zhao et al. (2022)
Temperature	Increased water temperature	Changes in water balance and chemistry degrade wetland functions in hydrological regulation and water purification.	Jolly et al. (2008)
	Elevated temperatures	Organisms’ physiological processes affected, reducing biodiversity and impairing freshwater wetland function.	Epele et al. (2022)
	Warmer temperatures	Climate warming affects plant adaptations, degrading nutrient cycling in FWs.	Lindborg et al. (2021)

### 2.1 Hydrology

Water plays a crucial role in the formation, development, succession, and extinction of wetlands, directly affecting their structure, function, and ecosystem stability (Wang et al., 2015). Human activities and climate change cause changes in precipitation, evapotranspiration, and temperature, which lead to changes in hydrological conditions such as water-holding capacity, water level, and inundation duration of wetlands (Karim et al., 2015). Changes in these hydrological characteristics in turn affect the structure, distribution (Todd et al., 2010; Maietta et al., 2020a) and biogeochemical cycling (Chen et al., 2013) of biological communities in FWs, leading to degradation of wetland ecosystem functions.

An increase in water loss from FWs leads to hydrological conditions variation and a decrease in available water resources, which can disrupt their freshwater supply (Zhao and Liu, 2016). Hydrological changes can also affect the structure, distribution and biogeochemical cycling of freshwater wetland biological communities, which in turn can degrade wetland ecosystems (Chen et al., 2013; Maietta et al., 2020a). Large fluctuations in water level can affect the structure and diversity of biological communities (Luo, 2009). During periods of low water levels in the Paraná River delta, the beta diversity and individual biomass of zooplankton decreases, leading to a simplification of the functional diversity (Gutierrez et al., 2022) and a degradation of the wetland environment that sustains aquatic vegetation in Lake Michigan-Lake Huron (DeVries-Zimmerman et al., 2021), whereas high water levels have led to a decrease in vegetation cover in Lake Ontario (Smith et al., 2021), resulting in habitat loss and the frustration of the supply functions of FWs. Overall, water level with too low or high is not conducive to wetland ecosystems. Soil water content, aeration conditions and redox potential also change with fluctuations in wetland water level, affecting the ecological processes and metabolic activities of microbial communities (Ma et al., 2018). Therefore, the relative stability of water level plays an important role in maintaining the functional stability of FWs.

### 2.2 Water quality and soil properties

Humans production and life discharge heavy metals (Li et al., 2021), pesticides and nutrient salts (Sremacki et al., 2020; Ding et al., 2021) into freshwater wetland ecosystems, directly and indirectly leading to changes in water quality and soil physicochemical properties of wetlands, which in turn cause wetland degradation (Wei et al., 2019). Relevant studies have shown that increased loading of nutrients such as nitrogen and phosphorus in water will deteriorate water quality, and cause eutrophication of the water body, leading to significant changes in the structure and function of wetland ecosystems (Khan and Ansari, 2005; Bano et al., 2022). It has been found that increased loading of nitrogen and phosphorus in FWs may affect the rates of nitrification, denitrification, and methane production, which in turn affects the nutrient cycling (Herbert et al., 2020). Soil physicochemical properties are key factors in shaping microbial community structure, composition, and metabolic activity (Ou et al., 2019). Changes in soil physicochemical properties caused by human disturbances and natural processes likewise have serious impacts on freshwater wetland biological communities (Lai, 2010).

### 2.3 Temperature

Temperature is recognized as one of the key climatic factors influencing the functional stability of FWs (Bano et al., 2022). Changes in temperature can have pervasive effects on the structure and function of freshwater wetland ecosystems (Hamilton, 2010). Wetland plant growth and photosynthesis efficiency increase with increasing temperatures within a certain range, increasing nutrient uptake and conversion (Zou et al., 2014). However, excessively high temperatures may reduce the germination of plant seeds and incubation of animals, which can have serious effects on wetland plant and microbial communities, disrupting wetland biodiversity (Nielsen et al., 2015). Temperature changes can also have an impact on microbial metabolism, for example, the role of iron-reducing bacteria in inhibiting methane production may diminish as the global average temperature increases, thus affecting greenhouse gas emissions from FWs. In addition, temperature changes may also lead to species migration and range shifts (Chen X. et al., 2023).

The hydrological conditions of wetlands are closely related to temperature changes, and global warming will lead to changes in evaporation and precipitation, which may alter the hydrological cycle of wetlands and thus indirectly affect the functional stability of wetlands (Luo, 2009; You et al., 2015). A previous study showed that a 10% decrease in rainfall will lead to changes in the redox conditions of the soil in the Everglades, thus affecting its biogeochemical processes; whereas the elemental load of the wetland ecosystem may increase when rainfall increases by 10%, which helps to maintain suitable redox conditions and promotes biogeochemical elemental cycling (Orem et al., 2015).

## 3 Impact of plant diversity on functional stability of freshwater wetlands

**Figure 1:**
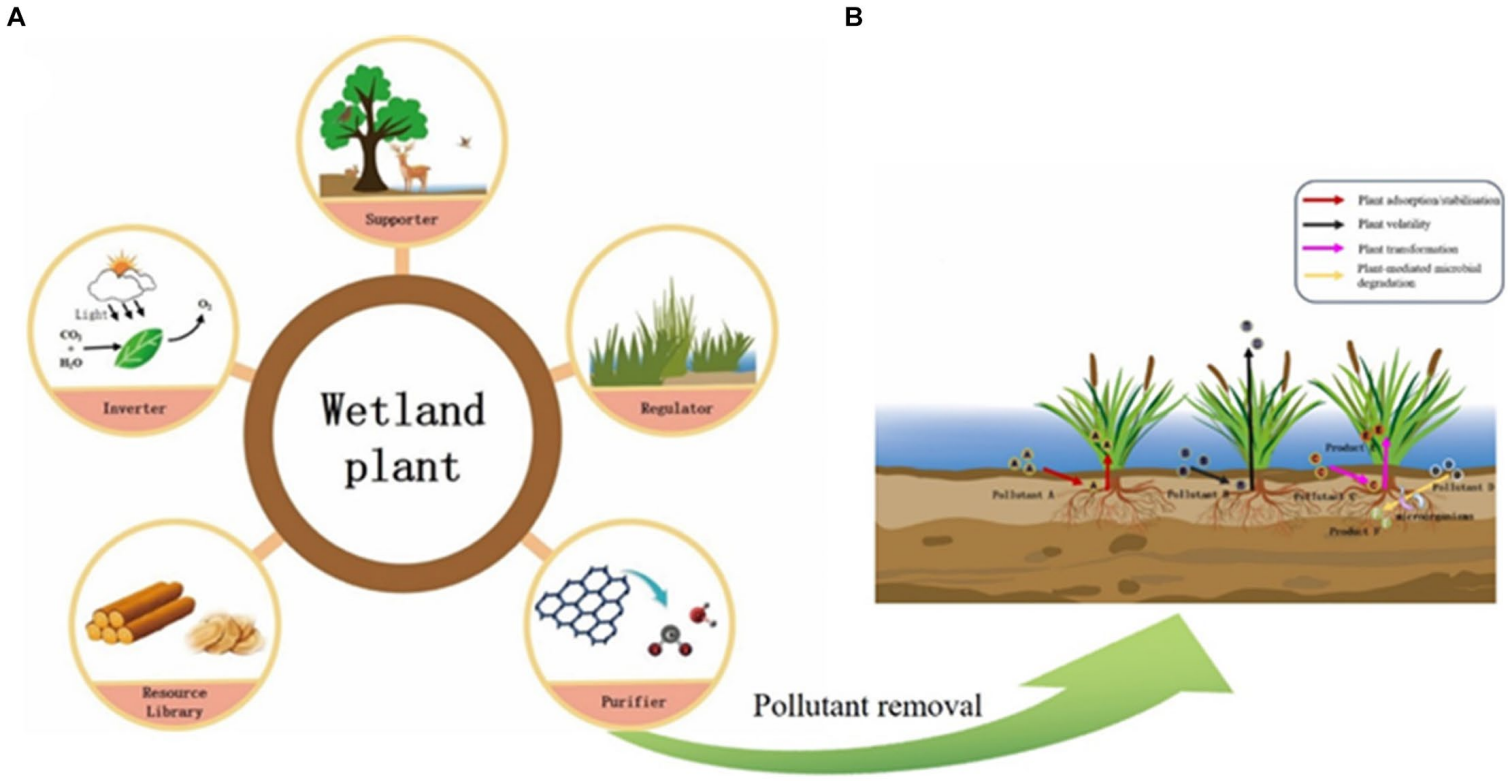
Role **(A)** and pollutant removal **(B)** of wetland plants.

Freshwater wetlands are rich in plant species, which play multiple roles in wetland ecosystems (Figure 1A). Different types of wetlands have different dominant vegetation, and diverse plants play an important role in maintaining the stability of wetland habitat functions (e.g., water purification, carbon storage, biodiversity maintenance, etc.) (Zhang et al., 2014).

### 3.1 Water purification

Removal of pollutants by wetlands plants is one of the main ways of water quality purification, mainly through two main pathways involving in direct pollutants removal and microbial processes mediating (Figure 1B; Stottmeister et al., 2003). The uptake of nutrients and heavy metals varies among different plant species (Adhikari et al., 2011; Abbasi et al., 2018). The study showed that the nitrogen uptake and fixation capacity of *Rhododendron ilfescens Siberianum* was higher, and the remediation of nitrogen pollution in wetlands was more effective (Weragoda et al., 2012). In addition, the dissolved oxygen in the water were affected by the abundance of submerged plant species (Qian, 2019), and different plants had different inter-roots, physiological processes, and growth modes, which might affect the community structure and activity of microorganisms, and further affect water quality purification (Zhang et al., 2010; Pang et al., 2016). Resource complementarity between plant species may also play a positive role in nutrient uptake and water purification (Choudhury et al., 2018). Therefore, maintaining high plant diversity can help to improve pollutants removal from water (Brisson et al., 2020).

### 3.2 Carbon storage

Freshwater wetlands are one of the valuable carbon storage sites, covering about 6% of the land area, and contain more than 30% of the soil carbon pool (Stewart et al., 2024). Plants play an important role in wetland carbon storage (Sheng et al., 2021). Wetland plants can convert atmospheric carbon dioxide into biomass through photosynthesis, and plant residues and leaves are deposited at wetland after death, which is one of the main mechanisms of carbon storage in wetlands (Adhikari et al., 2009). Previous studies have shown that the plants vary in nutrient and light utilization (Abbasi et al., 2018). Plant diversity has an important effect on freshwater wetland productivity (Isbell et al., 2013; Chaturvedi and Raghubanshi, 2015). Means et al. (2016) found a positive correlation between plant diversity and productivity in freshwater artificial wetlands. Cardinale et al. (2011) found that high diversity plant communities can use more ecological niches and increase the efficiency of nutrient utilization, which in turn increases primary productivity. An increase in wetland productivity can increase the capacity and total amount of carbon input from plants to the soil, which in turn increases carbon storage (Zhang et al., 2022).

In addition, the decomposition mode (humification and mineralization) and rate of plant apoplasts are particularly important for wetland carbon storage (Prescott and Vesterdal, 2021). Litter from different types of plants has different chemical compositions (Yan et al., 2018) and decomposition rates (Xi et al., 2023). It has been shown that the litter of freshwater wetland vegetation has the ability to alter the nutrient content of soil nitrogen and carbon, thus leading to the construction of different dominant microorganisms (Bonetti et al., 2021). Some plant litter leads to the production of microbial communities of humification, while others lead to the construction of microbial communities of carbon dioxide or methane production (Lin et al., 2015). Increased plant diversity can provide a wider variety of little, and this little can lead to the construction of more stable and resilient microbial communities, affecting the carbon storage capacity of the wetland (Maietta et al., 2020b).

### 3.3 Biodiversity maintenance

Plants can create unique microhabitat structures and provide suitable conditions for many animals and microorganisms (Choi et al., 2014; Weilhoefer et al., 2017). Freshwater wetland plants serve as the basis of the food chain in this ecosystem, and rich wetland plant communities provide a more complex and stable food web that supports the nutrient needs of many animals and microorganisms, thus contributing to the maintenance of biodiversity (Peel et al., 2019). In addition, higher plant diversity improves the resistance of wetland ecosystems to invasive alien species and better defends against invasive alien species, thus maintaining the stability of other organisms within the wetland (Peter and Burdick, 2010). Therefore, the protection and maintenance of plant diversity in FWs is essential for maintaining wetland biodiversity.

## 4 Impact of microbial diversity on functional stability of freshwater wetlands

Microorganisms in FWs are rich and diverse, with some differences in microbial composition among different wetland types, which can be mainly categorized into bacteria, archaea, fungi and protozoa (Cao et al., 2017). Microorganisms play an irreplaceable role in maintaining the stability of freshwater wetland habitat functions (e.g., water purification and biogeochemical cycles, etc.) (De Mandal et al., 2020; Chen M. et al., 2023; Qiao et al., 2023; Chen et al., 2024).

### 4.1 Water purification

Microorganisms can participate in various water purification processes through a series of metabolic and interaction processes, especially some functional microorganisms play a crucial role in wetland water purification (Wang et al., 2022). For example, some inter-root microorganisms such as *Pseudomonas* and *Flavobacterium* can effectively remove micropollutants (Brunhoferova et al., 2022). *Fusobacterium*, *Rhizobium* and *Erythrobacterium* have significant removal effects on organic pollutants such as petroleum in wetlands, and their removal rates are positively correlated with the abundance of bacterial species (Xiang et al., 2020). *Burkholderia*, *Hydrophilus*, and *Thiobacillus* play important roles in the remediation of arsenic and antimony pollution in wetlands (Deng et al., 2022).

The areas riched in wetland microbial diversity usually have higher degradation capacity of organic pollutants, and different microbial communities can co-operate together to decompose complex organic matter and convert it into harmless products (Berrier et al., 2022). Studies have shown that hydrocarbon-degrading microorganisms (e.g., *Pseudomonas*, *Rhodococcus*, and *Nocardia*) in FWs can form microbial aggregates, improving the removal efficiency of n-alkanes and polycyclic aromatic hydrocarbons (PAHs) (Liu et al., 2021). Anaerobic ammonia-oxidizing bacteria in wetlands can cooperate with certain archaea (e.g., nitrate archaea and sulfate-dependent archaea) to complete the denitrification process in wetlands (Wang et al., 2019). In addition, some microorganisms can remove multiple pollutants simultaneously. For example, *Flavobacterium* and *Chryseobacterium* can simultaneously degrade nitrogen and organic matter in wetlands (Shen et al., 2018). Sulfate-reducing bacteria, such as *Desulfovibrio*, *Desulfobacter*, and *Desulfobulbus*, also play dual roles in wetland restoration: (1) participating in the sulfate reduction process, producing hydrogen sulfide; (2) hydrogen sulfide reacts with heavy metals to form precipitation, which promotes the passivation of heavy metals (Chen et al., 2021).

### 4.2 Biogeochemical cycles

Wetland microorganisms are involved in the process of storage, transformation and release of C, N and other elements, and are the dominant driver of the biogeochemical cycle in FWs (Hussain et al., 2023).

**Figure 2:**
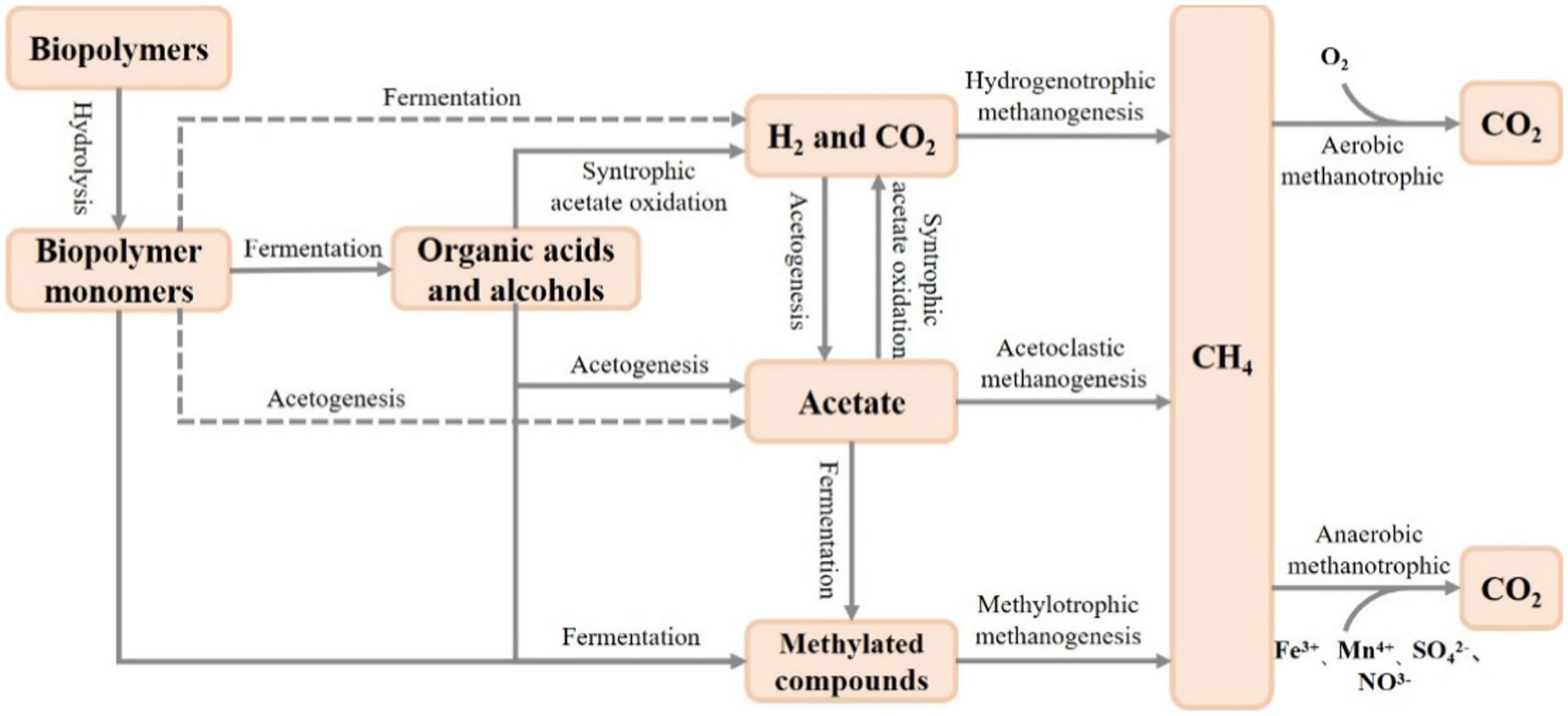
Methane production and transformation by wetland microorganisms.

The biogeochemical cycle of carbon in FWs has received much attention (Zou et al., 2022; Bao et al., 2023; Qian et al., 2023), and microorganisms are mainly involved in the carbon cycle through the processes of respiration, methane production and conversion, and decomposition of organic matter (Bardgett et al., 2008). Microorganisms play an important role in methane production and transformation of FWs (Figure 2). It is now widely accepted that methanogenic bacteria are distributed in seven orders of the phylum Euryarchaeota (Methanopyrales, Methanococcales, Methanobacteriales, Methanomicrobiales, Methanomassiliicoccales, Methanosarcinales, and Methanocellales) (Dean et al., 2018). Among them, Methanomicrobiales, Methanosarcinales, Methanomassiliicoccales, and Methanobacteriaceae methanogenic bacteria have widely found in wetland ecosystems (Horn Marcus et al., 2003; Zhang et al., 2008; Söllinger et al., 2016). There are three main pathways of freshwater wetland methanogens involved in methanogenesis: acetate fermentation, hydrogenotrophic and methylotrophic methanogenesis (Narrowe et al., 2019), whereas wetland methane oxidation is of two types: aerobic and anaerobic oxidation. The diverse microorganisms can adapt to the different environmental conditions and can better maintain the balance of wetland methane production and conversion. It was found that the microbial community can change the methanogenic pathway by adjusting the composition and activity of the microbial community under the fluctuation of nutrients, and then maintaining the stability of carbon cycle (Holmes et al., 2014). In addition, the richness of microbial diversity in FWs is closely related to the rate of mineralization of organic matter, and an active microbial community can increase organic matter degradation and mineralization (Li et al., 2015).

Microorganisms in FWs are also critical for maintaining the relative stability of the nitrogen cycle, and diverse microorganisms are an important player in driving nitrogen conversion and its cycling processes (Mellado and Vera, 2021; Sheng et al., 2023). Microorganisms such as nitrogen-fixing bacteria and cyanobacteria can convert atmospheric N_2_ into bioavailable forms such as ammonia and nitrate, supplying the wetland ecosystem with available nitrogen (Bae et al., 2018). It has been found that the efficiency and rate of nitrogen fixation are usually positively correlated with the number and diversity of microorganisms such as nitrogen-fixing bacteria (Li H. et al., 2022). On the other hand, some microorganisms (e.g., anaerobic ammonia-oxidizing bacteria, ammonia-oxidizing archaea, and denitrifying anaerobic methane-oxidizing bacteria) are also present in FWs, involved in key nitrogen transformation processes such as ammonia oxidation, nitrification and denitrification (Chen et al., 2020). These microorganisms differ in their tolerance and sensitivity to environmental factors, and a high diversity of microorganisms can provide different kinds of microbial functional groups, improving the adaptability and stability of FWs to environmental changes and maintaining the relative stability of the nitrogen cycle (Hu et al., 2017).

## 5 Impacts and mechanisms of habitat change on biodiversity

**Figure 3:**
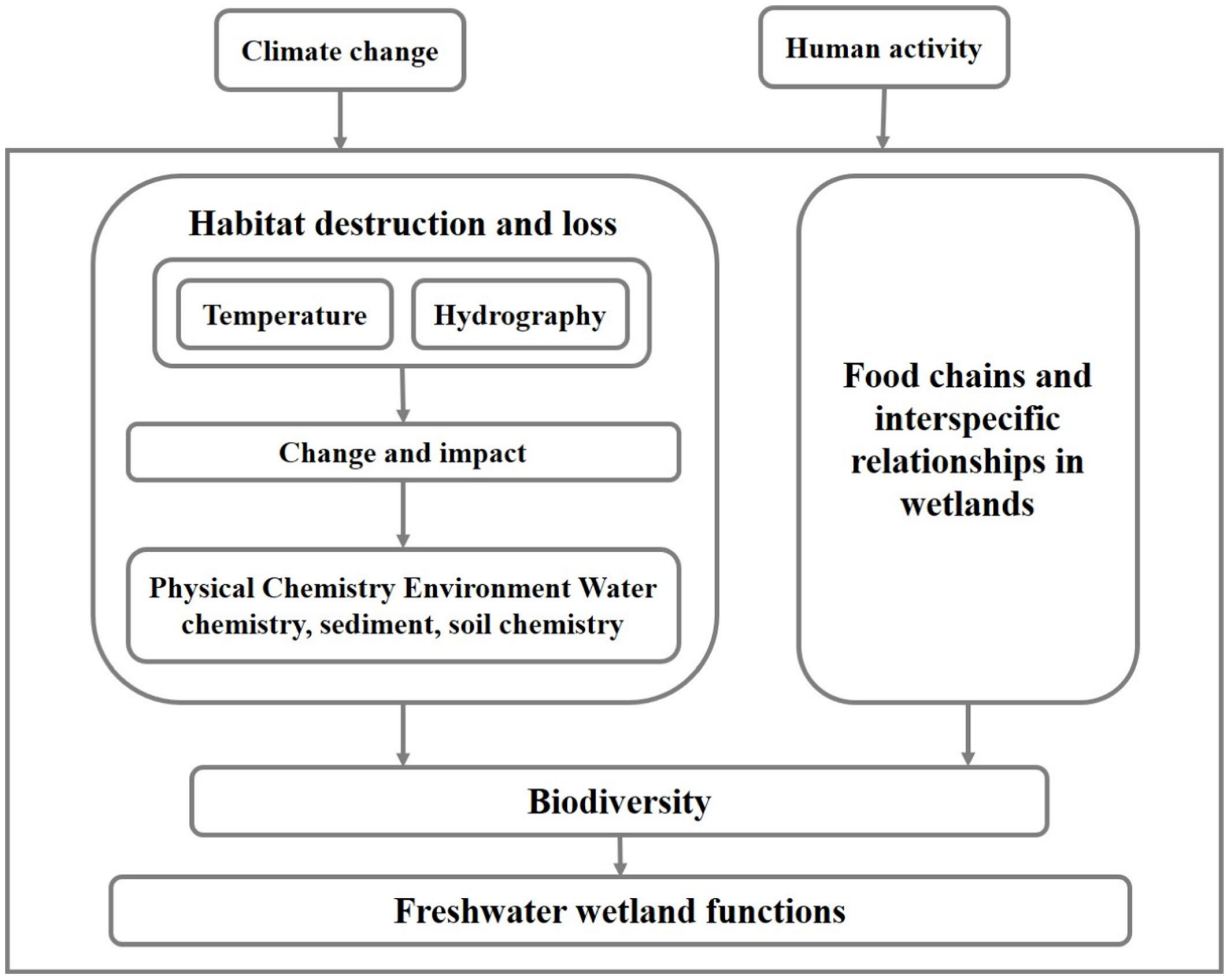
Impacts of habitat change on biodiversity in FWs.

Wetlands provide habitat for nearly 20% of the world’s species and are one of the most biodiversity-rich systems, however, they are under great pressure from human activities and climate change (Fang et al., 2006). This is causing a large degree of degradation of FWs and affecting the biodiversity of ecosystems (Al-Obaid et al., 2017). Habitat changes have important effects on wetlands (Figure 3). Among these, habitat changes and alterations in food chains and interspecific relationships are the two main factors (Ohba et al., 2019; Wang et al., 2021).

Habitat loss and fragmentation can result in the reduction and fragmentation of freshwater wetland areas, weakening the available area and connectivity of habitats for species, and these can directly lead to the reduction of the number and distribution range of some species, and consequently the decline of biodiversity (Jamin et al., 2020). For example, the size and connectivity of wetlands in Xin Jiang Wan Town, Shanghai, decreased with the accelerated urbanization of the area, leading to habitat loss and diversity reduction of wetland birds (Xu et al., 2018). Vascular plants in the wetlands of the canton of Zurich in eastern Switzerland became extinct as a result of the reduction of wetland connectivity and patch size under human activities (Jamin et al., 2020). In addition, the movement and migration of amphibians are limited when wetlands are fragmented, which may lead to the delayed extinction of these species (Gimmi et al., 2011).

Habitat change also affects wetland biodiversity by altering wetland food chains and interspecific relationships (Araújo et al., 2014). Previous studies have found that species richness of insectivorous birds in the Lampertheimer Altrhein area has decreased, due to the reducing food resources for insectivorous birds under agricultural intensification (Schrauth and Wink, 2018). The reduction in species richness and cover of plant communities during the degradation of the Ruoerge wetland has led to changes in the trophic structure of omnivores and algae, which in turn had a serious impact on the diversity of nematode communities (Wu et al., 2017). In addition, biological invasions are recognized as one of the main drivers of biodiversity loss (Mazor et al., 2018). Habitat changes can promote the invasion and spread of non-native species (e.g., *Spartina alterniflora*), and these invasive species can disrupt the original food chains and interspecific relationships of ecosystems, thus leading to biodiversity reduction (Wang et al., 2021).

In addition, changes in environmental factors such as wetland water level and pollution have significant impacts on biodiversity. For example, during the degradation of wet marshes to meadows in the Sanjiang Plain, changes in wetland water level alter the living conditions of organisms, which in turn affects the diversity and community composition of plants and microorganisms (Sui et al., 2017; Liping et al., 2020). The overuse of herbicides and pesticides in agricultural production activities has caused severe pollution of the Infranz wetlands in north-west Ethiopia, adversely affecting their biodiversity (Eneyew and Assefa, 2021).

## 6 Future prospects

Freshwater wetlands with high biodiversity play an extremely important role in maintaining the functional stability of wetland habitats. Many environmental drivers such as water level, water quality, soil properties, temperature, and biological drivers (e.g., plant/microbial diversity) have important impacts on the functional stability of freshwater wetland ecosystems, but many in-depth studies are needed in the following aspects in the future:

1. Changes in biodiversity can directly or indirectly regulate ecosystem processes, and biodiversity is the main determinant of maintaining ecosystem functional stability. Therefore, it is of great significance to investigate the relationship between biodiversity and functional stability. Nowadays, most studies on the functional stability and biodiversity of freshwater wetland have focused on small-scale scales and homogeneous habitats, ignoring the effects of spatial and temporal scales and environmental heterogeneity. Therefore, the study on the multi-scale integration and relationship between biodiversity and functional stability at different scales is important. This will help maintain the stability of freshwater ecosystems and provide theoretical support for the conservation of FWs.
2. Many studies are about the response of habitat function to environmental and biological elements in the context of global change. Most studies agreed that high levels of biodiversity can better maintain the stability of habitat function. In addition, changes in environmental factors can indirectly affect ecosystem habitat function through biodiversity. Therefore, future research needs to focus on the mechanisms by which environmental and biological factors drive habitat function enhancement through community composition, species diversity, environmental heterogeneity and biological interactions.

## 7 Conclusion

Freshwater wetlands are one of the most biodiverse ecosystems, and abundant species has a significant impact on the habitat function of FWs. Many environmental factors are changing under global change and human activities, and these changes can either directly affect the stability of wetland habitat functions or indirectly affect habitat functions by altering the biodiversity of FWs. Our study analyzes the roles of environmental drivers maintaining the stability of wetland habitat functions, such as hydrology, temperature, and water quality, discusses the impacts of plant and microbial diversity on the functional stability of FWs, and further reveals the impacts and mechanisms of habitat changes on biodiversity. In general, biodiversity can promote the stability of habitat functions in FWs. However, most studies focus on small-scale scales and homogeneous habitats. Therefore, future studies on biodiversity and stability of habitat functions in FWs at large scales and non-homogeneous habitats still need to be further explored.

## Author contributions

AS: Writing – original draft. SL: Data curation, Software, Validation, Writing – review & editing. HL: Conceptualization, Project administration, Supervision, Writing – review & editing. BY: Writing – review & editing.
